# Interactions between integrated pest management, pollinator supplementation, and normalized difference vegetation index in pumpkin, *Cucurbita maxima* (Cucurbitales: Cucurbitaceae), production

**DOI:** 10.1093/ee/nvad035

**Published:** 2023-05-12

**Authors:** Nduta A Waithaka, Muo Kasina, Namikoye E Samita, Mary M Guantai, Evanson R Omuse, Nadia K Toukem, H Michael G Lattorff, Elfatih M Abdel-Rahman, Marian Adan, Samira A Mohamed, Thomas Dubois

**Affiliations:** Department of Agricultural Science and Technology, Kenyatta University, P.O. Box 43844-00100, Nairobi, Kenya; Apiculture and Beneficial Insects Research Institute (ABIRI), Kenya Agricultural and Livestock Research Organization (KALRO), P.O. Box 32-30403, Marigat, Kenya; Apiculture and Beneficial Insects Research Institute (ABIRI), Kenya Agricultural and Livestock Research Organization (KALRO), P.O. Box 32-30403, Marigat, Kenya; Department of Agricultural Science and Technology, Kenyatta University, P.O. Box 43844-00100, Nairobi, Kenya; Kenya Plant Health Inspectorate Service (KEPHIS), P.O. Box 19164-00501, Nairobi, Kenya; International Centre for Insect Physiology and Ecology (icipe), P.O. Box 30772-00100, Nairobi, Kenya; Department of Zoology and Entomology, University of Pretoria, Hatfield, 0028 Pretoria, South Africa; International Centre for Insect Physiology and Ecology (icipe), P.O. Box 30772-00100, Nairobi, Kenya; International Centre for Insect Physiology and Ecology (icipe), P.O. Box 30772-00100, Nairobi, Kenya; International Centre for Insect Physiology and Ecology (icipe), P.O. Box 30772-00100, Nairobi, Kenya; International Centre for Insect Physiology and Ecology (icipe), P.O. Box 30772-00100, Nairobi, Kenya; International Centre for Insect Physiology and Ecology (icipe), P.O. Box 30772-00100, Nairobi, Kenya

**Keywords:** *Cucurbita maxima*, integrated pest and pollinator management (IPPM), *Hypotrigona* sp, *Zeugodacus cucurbitae*

## Abstract

Sustainable production of pumpkin (*Cucurbita maxima* Duchesne) partly relies on integrated pest management (IPM) and pollination services. A farmer-managed field study was carried out in Yatta and Masinga Sub-Counties of Machakos County, Kenya, to determine the effectiveness of a recommended IPM package and its interaction with stingless bee colonies (*Hypotrigona* sp.) for pollinator supplementation (PS). The IPM package comprised Lynfield traps with cuelure laced with the organophosphate malathion, sprays of *Metarhizium anisopliae* (Mechnikoff) Sorokin isolate ICIPE 69, the most widely used fungal biopesticide in sub-Saharan Africa, and protein baits incorporating spinosad. Four treatments—IPM, PS, integrated pest and pollinator management (which combined IPM and PS), and control—were replicated 4 times. The experiment was conducted in 600 m^2^ farms in 2 normalized difference vegetation index (NDVI) classes during 2 growing seasons (October 2019–March 2020 and March–July 2020). Fruits showing signs of infestation were incubated for emergence, fruit fly trap catches were counted weekly, and physiologically mature fruits were harvested. There was no effect of IPM, PS, and NDVI on yield across seasons. This study revealed no synergistic effect between IPM and PS in suppressing Tephritid fruit fly population densities and damage. *Hypotrigona* sp. is not an efficient pollinator of pumpkin. Therefore, we recommend testing other African stingless bees in pumpkin production systems for better pollination services and improved yields.

## Graphical Abstract

**Graphical Abstract:**
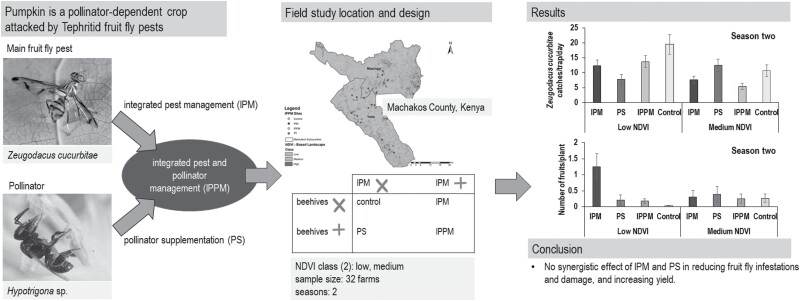

## Introduction

Pumpkins, *Cucurbita maxima* Duchesne (Cucurbitales: Cucurbitaceae), produce fruits and leaves that are rich in proteins, carbohydrates, and oils containing unsaturated fatty acids (Indrianingsih et al. 2019). Their seeds contain minerals such as nitrogen, phosphorous, sodium, potassium, calcium, magnesium, iron, copper, zinc, and manganese (Nawirska et al. 2009). In Kenya, pumpkins are mainly grown by smallholder farmers for household use, while the surplus is traded for cash income (Machakos County Integrated Development Plan 2019). Pumpkins, therefore, provide affordable nutritious food to low-income families and curb food insecurity (Rődlach 2011). Production in the country is still low compared with neighboring countries, with the area under pumpkin production at 1,293 ha and 1,380 ha in 2017 and 2018, respectively (Horticultural Crops Directorate 2018). In Kenya, pumpkin production mainly occurs in semiarid areas, which comprise the largest area of arable land in the country (Ndengwa et al. 2016). Machakos County is a major production area, where households prefer fruit to leaf consumption (Ndegwa et al. 2016).

Pumpkin production in Kenya is seriously constrained by pests, including the silverleaf whitefly, *Bemisia tabaci* Gennadius (Hemiptera: Aleyrodidae) (Messelink et al. 2008), and several species of Tephritid fruit flies (Diptera: Tephritidae) (De Meyer et al. 2012, Kambura et al. 2018). Tephritid fruit flies seem to be the most destructive pests of pumpkin fruits, particularly the melon fruit fly, *Zeugodacus cucurbitae* Coquillett (Diptera: Tephritidae), which can cause up to 100% yield loss without adequate control measures (Mwatawala et al. 2006, Deguine et al. 2012, Badii et al. 2015, Doorenweerd et al. 2018). In Kenya, this pest has been reported to cause very high yield losses to cucurbits, particularly pumpkin fruits (Kambura et al. 2016). *Zeugodacus cucurbitae* occurrence is directly influenced by abiotic factors, particularly temperature, which affect its development, survival, and distribution (Fletcher 1989, Aluja et al. 2014, Hill et al. 2016). Other important Tephritid fruit fly species associated with cucurbits include the greater pumpkin fruit fly, *Dacus bivittatus* Bigot; the lesser pumpkin fly, *Dacus ciliatus* Loew; the jointed pumpkin fly, *Dacus vertebratus* Bezzi; and the tomato fruit fly, *Dacus punctatifrons* Karsch (Okolle and Ntonifor 2005, Kambura et al. 2016, Layodé et al. 2019). The host preference of the different fruit fly species affecting cucurbits varies according to the environment, availability of host plants, and other competing pests (Charlery et al. 2017).

Crop production, including pumpkin, is highly influenced by the agricultural landscape, which affects the distribution, diversity, and abundance of pest and beneficial arthropods because of their interaction with abiotic factors such as temperature, altitude, and rainfall (O’Rourke and Jones 2011). The normalized difference vegetation index (NDVI) has been widely used to understand landscape and vegetation phenology. Recent studies have reported the use of NDVI as a proxy for green biomass, which is related to canopy photosynthesis (Wang et al. 2017) and vegetation biodiversity (Beck et al. 2007). Vegetation biodiversity at landscape scales is key as it influences population densities of both pollinators and natural enemies, which use available resources in alternative habitats for their development (Shackelford et al. 2013). The abundance of natural enemies particularly may in turn influence pest population in the ecosystem (Copeland et al. 2006). Studies have reported how NDVI can predict the distribution and abundance of insect pests and pollinators and monitor long-term landscape structure (Abdel-Rahman et al. 2017, Toukem et al. 2020, 2022). For example, Toukem et al. (2020) reported a high density of Tephritid fruit flies infesting avocado, *Persea americana* Miller (Laurales: Lauraceae), in low NDVI. In this study, NDVI was used as a proxy for landscape vegetation.

Tephritid fruit flies in cucurbit production systems are usually controlled by chemical insecticides (Dhillion et al. 2005), which have negative effects on human, animal, and environmental health, including pollinators, and may cause the development of insecticide resistance (Cloyd and Bethke 2015, Nobre et al. 2019). Furthermore, misuse of insecticides may lead to surpassing allowed residue limits and raising trade challenges (Badii et al. 2015, Mebdoua 2018). To reduce reliance on chemical pesticides, integrated pest management (IPM) approaches are being researched and deployed for Tephritid fruit fly control in Africa, largely on mango, *Mangifera indica* Linnaeus (Sapindales: Anacardiaceae) (Korir et al. 2015, Muriithi et al. 2016, 2020). The use of cultural practices, particularly field sanitation whereby infested fruits are removed, is commonly used for fruit fly management in Africa and can significantly reduce Tephritid fruit fly populations and minimize yield losses (Niassy et al. 2022). Sterile insect technique, which suppresses the pest population by maintaining a barrier of sterile male flies, has been used successfully in Japan against the melon fruit fly (Ito et al. 2003). Male annihilation technique consists of the deployment of high-density trapping stations with male-specific lures combined with an insecticide. The use of cuelure (the pheromone 4-(p-acetoxyphenyl)-2-butanone, which is highly attractive to male *Z. cucurbitae*) laced with fipronil, has been used successfully against *Z. cucurbitae* and fruit fly species in genera *Bactrocera* (Spafford et al. 2018). Among the biopesticides used in sub-Saharan Africa, *Metarhizium anisopliae* (Mechnikoff) Sorokin (Hypocreales: Clavicipitaceae) isolate ICIPE 69 (Real IPM, Thika, Kenya) is widely used as a fungal biopesticide (Akutse et al. 2020) and has been found to be highly potent against *Z. cucurbitae* (Onsongo et al. 2019). Protein baits, which are mainly female attractants, entail the application of food baits, combined with insecticides in localized spots against fruit flies, and have been effective in the management of *Z. cucurbitae* (Shikano et al. 2022). IPM against *Z*. *cucurbitae* has been developed and implemented across the Pacific regions (Vargas et al. 2015), but there are few reports on IPM practices for fruit fly management in pumpkin production systems in East Africa.

Pumpkin is a pollination-dependent crop (Vidal 2010), with the honeybee *Apis mellifera* Linnaeus (Hymenoptera: Apidae) reported as the most important pollinator for cucurbits in Kenya (Njoroge et al. 2010). Often, there is a pollination deficit in Africa, as demonstrated for avocado (Sagwe et al. 2021), which can be solved through pollination supplementation with honeybees (Sagwe et al. 2021). The study area in Machakos is characterized as a semiarid area with limited natural habitats (Ndengwa et al. 2016). Limitations in pumpkin pollination in the area may be attributed to the reduced natural habits, which have been reported to reduce species richness and diversity of pollinators (Carvalheiro et al. 2010). However, African stingless bees (Hymenoptera: Apidae) might offer a better alternative for cucurbit pollination, especially for smallholders because of their ecological adaptability, easy domestication, floral constancy, perennial colonies, and polylectic nature (Heard 1999). *Hypotrigona* sp. was the preferred pollinator for the present study. This parasitic wasp is widely distributed across the tropics, lives in large colonies (Kiatoko et al. 2012), is a generalist in terms of food and nesting resources, and has been reported to pollinate other cucurbits such as the greenhouse cucumber *Cucumis sativus* Linnaeus (Cucurbitales: Cucurbitaceae), yielding 90% seed germination (Kiatoko et al. 2021b). The genus *Hypotrigona* contains several species (*Hypotrigona gribodoi* Magretti, *Hypotrigona araujoi* Michener, and *Hypotrigona ruspolii* Magretti in East Africa and *Hypotrigona penna* Eardley in West Africa) (Ndungu et al. 2017), which are reported to offer good pollination services in cucurbits (Kiatoko et al. 2021b). Studies carried out in Kenya have shown that *Hypotrigona* sp. successfully pollinate cucurbits such as cucumber and Galia muskmelon *Cucumis melo* L. var. reticulatus ser. (Cucurbitales: Cucurbitaceae) (Kiatoko et al. 2021a, 2021b). Other important African stingless bees that can be exploited for cucurbit pollination include *Meliponula ferruginea* Lepeletier (Hymenoptera: Apidae) and *Meliponula bocandei* Spinola (Hymenoptera: Apidae). These bees have been reported to successfully pollinate coffee *Coffea canephora* Pierre ex A. Froehner (Gentianales: Rubiaceae) in Uganda, with pollination efficiency of 98% and 95% fruit set, respectively (Munyuli 2010).

Integrated pest and pollination management (IPPM) is a holistic strategy that synergizes pest management practices and pollination services in an ecologically and economically sustainable manner (Biddinger and Rajotte 2015, Merle et al. 2021) especially in pollinator-dependent crops that are highly affected by damaging insect pests, such as cucurbits (Leach et al. 2022). IPPM focuses on managing pests while simultaneously mitigating the negative impact of control methods on pollinator health (Biddinger and Rajotte 2015). The aim of this study was, therefore, to determine the effect of IPM and pollinator supplementation (PS), and their interaction, on Tephritid fruit fly population densities and damage, and pumpkin yield and assess whether landscape vegetation influences these effects.

## Materials and Methods

### Study Site

**Fig. 1.**
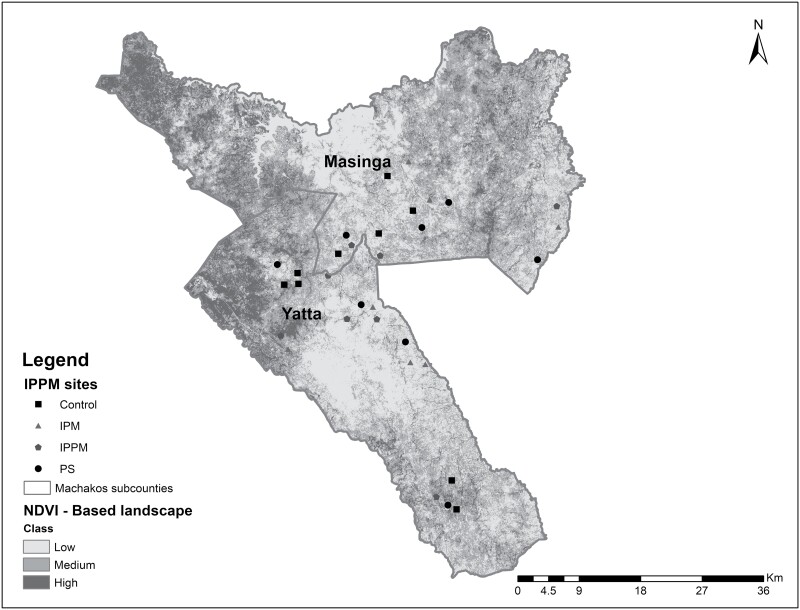
Distribution of pumpkin farms in Yatta and Masinga Sub-Counties, Machakos County.

The study was carried out in farmer-managed pumpkin fields in Yatta (1°24ʹ S, 37°59ʹ E) and Masinga Sub-Counties (0°58’ S, 37°36’ E) in Machakos County within low and medium normalized difference vegetation index (NDVI) classes (Fig. 1). Machakos County covers a total area of 6,208 km^2^ and lies at an elevation of 790–1,594 m above sea level. The County receives an average annual rainfall of between 500 and 1,250 mm, with October–November as the main rainy season (Machakos County Integrated Development Plan 2019).

### Computation of NDVI

Rainfall data from the Climate Hazard Group Infrared Precipitation with Station (CHIRPS, University of California, Santa Barbara, USA) were used at a 5 × 5 km spatial resolution to determine the exact dry and wet seasons in 2019 for the study area. Sentinel-2 (S2) satellite images were then utilized to compute the NDVI in the cloud-based Google Earth Engine platform (GEE, Alphabet, Mountain View, USA) according to Gorelick et al. (2017). One hundred and ninety-five S2 images were acquired for both the wet (February–May, 99 images) and dry (June–August, 96 images) seasons. We then created a composite image for each season using the multi-date S2 median reflectance pixel values at 10-m spatial resolution. A detailed description of the S2 image processing steps is found in Adan et al. (2021). Using Equation (1), we calculated the NDVI for both dry and wet composite images based on red and near-infrared bands.


$$\begin{array}{l} NDVI\;=\frac{\left(NIR-red\right)}{\left(NIR+red\right)} \end{array}$$ (1)

where NIR and red are reflectance values at near-infrared and red bands 8 and 4 of S2, respectively.

The wet and dry season NDVI values were reclassified into 3 vegetation productivity classes (low, medium, and high) using the unsupervised *K*-means clustering method (Adan et al. 2021). The ranges of NDVI values for each class for the dry season were as follows: 0.320–0.077 (low), 0.077–0.133 (medium), and 0.133–0.619 (high), while the NDVI ranges for the wet season were as follows: 0.373–0.158 (low), 0.158–0.249 (medium), and 0.249–0.730 (high). Subsequently, a multiseason composite classified map was created by combining the outputs of the wet and dry season classifications based on the cluster values for both seasons (Adan et al. 2021).

### Experimental Layout

Two experiments were carried out during the 2 pumpkin growing seasons (October 2019–March 2020 and March–July 2020). The first and second seasons are characterized by periods of long and short rains, respectively. The experiment consisted of 2 factors—(i) PS (absence or presence of managed *Hypotrigona* sp. colonies) and (ii) IPM (absence or presence of IPM)—conducted as a full-factorial design, yielding 4 treatments (control, PS, IPM, and IPPM, with IPPM representing the presence of both PS and IPM). A total of 32 farmer fields were included in the experiments. Each treatment was replicated 4 times in separate farmer fields, both in low and medium NDVI classes, yielding a total of 8 farmer fields per treatment (Fig. 1). Suitable farms were determined using a baseline survey carried out during July–August 2019. Treatments were allocated to pumpkin farmers based on their willingness to pay for the IPM, PS, or IPPM. Those willing to pay for both IPM and PS were randomly selected to host the IPPM treatment; those willing to pay for either IPM or PS only were randomly selected to host the IPM and PS treatments, respectively; and those not willing to pay for either package were randomly selected to host the control treatment. A minimal distance was kept among farms, depending on the treatment option, based on criteria that were developed and described by Adan et al. (2021). Specifically, (i) the distance between IPPM and PS farms was at least 1.5–3.0 km, (ii) the distance between IPM and control farms was at least 0.5 km, and (iii) the distance between IPPM or PS farms was at least 3.5 km away from either IPM or control. During the experiments, all plots were farmer-managed. Therefore, each farmer carried out similar agronomic practices such as land preparation, planting, weeding, and top-dressing with fertilizer independently. No plots were sprayed with insecticides during the entire period of the study.

### Planting and Application of IPM and PS Packages

In each field, plots measuring 20 × 30 m used for growing pumpkins were demarcated. Each farmer was supplied with 150 pumpkin seeds (Dora F1 Hybrid, Safari Seeds, Nairobi, Kenya), which were sown directly in the soil at a spacing of 2 × 2 m and a depth of 3–4 cm. Three weeks from sowing, the crops were top-dressed with 15 g/plant of 17-17-17 NPK fertilizer (DMBL Ruiru, Nairobi, Kenya).

The IPM package for this study comprised the following: (i) 2 Lynfield traps fitted with cuelure and cotton balls laced with the organophosphate malathion (Farmtrack Consulting, Nairobi, Kenya) were placed along a transect line in the middle of the farm running along the longest side of the farm. The traps were placed 50 m from each other and installed 8 weeks after crop germination (at the onset of flowering) (Toukem et al. 2020). A metallic stand was anchored 0.3 m into the ground and was used to hold the traps 1 m above the ground. The lures and malathion cotton balls in the traps were replaced 6 weeks after installation. (ii) One hundred milliliters protein bait made from brewer’s yeast (Fruit Fly Mania, Kenya Biologics, Makuyu, Kenya), laced with 0.2-ml spinosad (Tracer, Dow Chemical East Africa, Nairobi, Kenya) was added to a hand spray pump containing 900 ml of water, thoroughly mixed and applied weekly from the onset of flowering to harvesting. (iii) The fungal biopesticide *Metarhizium anisopliae* isolate ICIPE 69 (Mazao Campaign, Real IPM) was applied once at the onset of flowering by adding 10 ml of the product in 15 liters of water contained in a knapsack. Soil was drenched with the mixture on the entire plot. Unlike the IPM and IPPM plots where 2 Lynfield traps were installed, only one Lynfield trap baited with cuelure and malathion was installed in the control and PS plots to monitor fruit fly populations.

*Hypotrigona* sp. bee colonies for PS treatment were obtained from stingless beekeeping farms in Mwingi, Kenya. The colonies were contained in stingless bee-specific hives, which were small rectangle wooden boxes (30.45 × 8.50 × 7.59 cm). They were constructed by local carpenters using local wood, with all spaces sealed except for a tiny hole (10 mm diameter), which the bees used to create their entrance/exit. The top cover was held with hinges and tightly fixed to avoid open spaces. Stingless bees were collected from the wild and allowed to settle in the hives before transferring them to the trial sites. During transportation, care was taken to avoid workers escaping by sealing the entrance/exit holes with mud (Kiatoko, personal communication). Beehives were installed in farms hosting IPPM and PS treatments at a density of 2 hives/farm at the onset of flowering. The hives were hung on trees within each farm at the edge of the long side of the plot (no separation distance was maintained since the stingless bees do not attack people) and kept throughout the study period.

### Data Collection

Field data collection began 8 weeks after pumpkin germination and continued throughout the flowering and fruiting crop stages for 8 weeks. Fruit flies caught in the Lynfield traps were collected weekly in 75-milliliter plastic bottles and transported for further analysis to the entomology laboratory at National Sericulture Research Centre (NSRC), Kenya Agricultural and Livestock Research Organization (KALRO), Thika, Nairobi. Contents of the plastic bottles were emptied into 85 mm (diameter) × 15 mm (height) plastic Petri dishes containing 70% ethyl alcohol. Fruit flies were morphologically identified to species level according to the keys described by White (2006). The number of each species was recorded per sample. If in doubt, confirmatory morphological identification of the fruit flies was done at the International Centre of Insect Physiology and Ecology (*icipe*), Nairobi, Kenya by R. Copeland.

Pumpkin fruits that had detached from the plant and had symptoms of fruit fly infestation (presence of oviposition marks and larvae) were collected weekly from the field, placed in 30 × 21 cm brown paper bags, and transported to NSRC. Damaged fruits were counted, weighed individually, and incubated. Incubation containers varied based on the size of the fruits: small fruits were incubated in 2-liter plastic containers, medium-sized fruits were incubated in 11-liter plastic containers, and large fruits were incubated in 20-liter plastic containers. All containers were lined with 305 × 305 mm serviettes and the rims were covered with 0.1 µm (pore size) muslin nets held in place with rubber bands. The containers were checked daily, and emerged puparia were collected, counted, and placed in 85 (diameter) × 15 mm (height) Petri dishes for emergence. Emerged adults were identified based on the above procedures and their counts recorded.

On a weekly basis, the health of the hives was confirmed by verifying adequate numbers of worker bees leaving and entering the hives. Absconded hives were replaced. Sixteen weeks after crop germination (at maturity), all pumpkin fruits were harvested once in all plots. The number of healthy and damaged harvested fruits/plant and the weight/fruit were recorded.

### Data Analysis

Fruit fly abundance was expressed as daily catches/trap/farm before analysis. Infestation of pumpkin fruits was expressed as the number of puparia per kilogram prior to analysis. Yield was expressed in kilogram per hectare. Trap catches for *Z. cucurbitae*, *D. bivittatus*, and *D. punctatifrons* were [log_10_(*x* + 1)] transformed to obtain a normal distribution with equal variance and subjected to a linear mixed-effect model to analyze the effects of IPM, PS, NDVI, and interactions, with the farm included as a random factor. However, very low catches of *Bactrocera dorsalis* Hendel (Diptera: Tephritidae), *D. vertebratus*, and *D. ciliatus* were obtained from trap catches; hence, they were not considered for subsequent analyses. The performance of the models was assessed by inspecting residual error distribution and checking for autocorrelation. A correlation structure was included in the model when there was evidence of autocorrelation (Durbin Watson test statistic < 1 or > 3). Wald chi-square tests were used to test fixed effects in the models, and when significant, means were separated using a Tukey HSD test. We performed all mixed models using the *lme4* package (Bates et al. 2015), with the *lmer* function. However, the number of puparia per kilogram did not assume a normal distribution and was analyzed using the aligned rank transformation analysis of variance (ANOVA) to assess the effects of IPM, PS, NDVI, and interactions. The direct effect of IPM, PS, NDVI, and their interactions on the yield and number of fruits per plant were assessed using a linear model. All analysis was performed in R software version 4.0.5 (R Core Team 2020), and the significance was assessed at 5%.

## Results

### Fruit Fly Trap Catches

Three fruit fly taxa belonging to the genera *Zeugodacus*, *Dacus*, and *Bactrocera* were identified from the trap catches. Fruit flies were identified as *Z. cucurbitae*, *D. punctatifrons*, *D. bivittatus*, *D. vertebratus*, *D. ciliatus*, and *B. dorsalis.*

During the first season, there was no significant effect of either IPM, PS, or NDVI, or their interaction (χ^2^ ≤ 0.98, df = 1, 246, *P* ≥ 0.37) on the daily catches of *Z. cucurbitae*, which averaged 5.76 ± 0.42 catches/trap/day (Table 1). There was a main effect of PS in the second season (χ^2^ = 5.19, df = 1, 246, *P* = 0.023), with densities without PS (12.51 ± 1.17 catches/trap/day) higher than densities with PS (9.84 ± 0.90 catches/trap/day). The mean daily catches of *Z. cucurbitae* were not significantly different between farms with and without IPM (χ^2^ = 0.83, df = 1, 246, *P* = 0.36), or across NDVI (χ^2^ = 2.16, df = 1, 246, *P* = 0.14). In the second season, a significant interaction occurred among IPM, PS, and NDVI (χ^2^ = 4.82, df = 1, 246, *P* = 0.028), with the lowest densities recorded on IPPM farms in medium NDVI (5.41 ± 1.07 catches/trap/day) and the highest densities on control farms in low NDVI (19.47 ± 3.32 catches/trap/day).

**Table 1.** Model estimates (standard error) from linear mixed-effects models fitted to estimate effects of pest control (IPM) (no, yes), PS (no, yes), and NDVI (low, medium), on *Z*. *cucurbitae*, *D*. *bivittatus* and *D*. *punctatifrons* abundance in Machakos County, Kenya

	Z. cucurbitae		D. bivittatus		D. punctatifrons	
	Season 1	Season 2	Season 1	Season 2	Season 1	Season 2
(Intercept)	5.78***	12.16***	1.43***	1.71***	1.96***	1.29***
	(0.33)	(0.26)	(0.07)	(0.12)	(0.11)	(0.05)
IPM (yes)	−0.87	−0.71	−0.84	−0.86	−0.97	−0.99
	(0.47)	(0.37)	(0.09)	(0.17)	(0.15)	(0.08)
Stingless bee introduction (yes)	−0.75	−0.43*	−0.83	−0.71	−0.87	−0.86
	(0.47)	(0.37)	(0.09)	(0.17)	(0.15)	(0.08)
NDVI (medium)	−0.63	−0.58	−0.73**	−0.74	1.00	−0.96
	(0.47)	(0.37)	(0.09)	(0.17)	(0.15)	(0.08)
IPM (yes) × stingless bee introduction (yes)	1.02	2.76	1.58**	1.81*	1.11	1.08
	(0.66)	(0.53)	(0.13)	(0.24)	(0.21)	(0.11)
IPM (yes) × NDVI (medium)	1.83	1.25	1.26	1.21	1.37	−0.99
	(0.66)	(0.53)	(0.13)	(0.24)	(0.21)	(0.11)
Stingless bee introduction (yes) × NDVI (medium)	1.56	3.13*	1.28	1.81*	1.40	1.23
	(0.66)	(0.53)	(0.13)	(0.24)	(0.21)	(0.11)
IPM (yes) × stingless bee introduction (yes) × NDVI (medium)	−0.64	−0.20*	−0.59**	−0.43*	−0.74	−0.82
	(0.94)	(0.74)	(0.19)	(0.34)	(0.30)	(0.15)

Estimates were tested with the Wald χ^2^ test and appreciated at the 5% level of significance. Significance code: ***P < 0.001, **P < 0.01, *P < 0.05.

In the first season, the mean daily catches of *D. bivittatus* differed significantly across NDVI (χ^2^ = 10.87, df = 1, 246, *P* < 0.0001), with densities in low NDVI (0.43 ± 0.05 catches/trap/day) higher than densities in medium NDVI (0.10 ± 0.01 catches/trap/day) (Table 1). However, no significant effect of IPM (χ^2^ = 3.28, df = 1, 246, *P* = 0.070) or PS (χ^2^ = 3.76, df = 1, 246, *P* = 0.053) was observed. A significant interaction among IPM, PS, and NDVI (χ^2^ = 7.87, df = 1, *P* = 0.005) was also reported. In low NDVI, IPPM farms recorded the highest densities (0.73 ± 0.16 catches/trap/day), while the lowest densities were reported on control farms in medium NDVI (0.06 ± 0.24 catches/trap/day). In the second season, there was a main effect of PS on mean daily catches of *D. bivittatus* (χ^2^ = 4.08, df = 1, 246, *P* = 0.043) with higher densities on PS farms (0.64 ± 0.08 catches/trap/day) than on farms without PS (0.52 ± 0.05 catches/trap/day). There was a significant interaction among IPM, PS, and NDVI (χ^2^ = 6.20, df = 1, 246, *P* = 0.013) on the mean daily catches of *D. bivittatus*, with highest densities on IPPM farms in low NDVI (1.13 ± 0.22 catches/trap/day) and lowest densities on PS farms in low NDVI (0.25 ± 0.07 catches/trap/day).

During the first season, there was no significant effect of IPM, PS, NDVI, or their interaction on the daily catches of *D. punctatifrons* (χ^2^ ≤ 1.30, df = 1, 246, *P* ≥ 0.25), which averaged 1.38 ± 0.09 catches/trap/day (Table 1). In the second season, there was a main effect of PS on the daily catches of *D. punctatifrons* (χ^2^ = 4.09, df = 1, 246, *P* = 0.043) with higher densities on farms without PS (0.29 ± 0.03 catches/trap/day) than on PS farms (0.22 ± 0.05 catches/trap/day). However, no significant effects of IPM (χ^2^ = 0.00, df = 1, 246, *P* = 0.95) or NDVI (χ^2^ = 0.35, df = 1, 246, *P* = 0.56) were observed. There was no significant interaction among IPM, PS, and NDVI on *D. punctatifrons* catches in the second season (χ^2^ = 1.81, df = 1, 246, *P* = 0.18).

### Pumpkin Infestation

*Zeugodacus cucurbitae*, *D. bivittatus*, *D. vertebratus*, and *D. ciliatus* were recovered from incubated pumpkin fruits. Their population densities were lumped together since their emergences were very low.

In the first season, the number of puparia per kilogram of fruit did not differ significantly across IPM (*F* = 0.28, df = 1, 248, *P* = 0.60), PS (*F* = 0.24, df = 1, 248, *P* = 0.62), or NDVI (*F* = 0.21, df = 1, 248, *P* = 0.65) (Table 2). In the second season, IPM significantly (*F* = 4.88, df = 1, 248, *P* = 0.028) influenced pumpkin fruit infestation. There was, however, no main effect of NDVI (*F* = 3.57, df = 1, 248, *P* = 0.060) and PS (*F* = 0.16, df = 1, 248, *P* = 0.69) on infestation. A significant interaction among IPM, PS, and NDVI was observed (*F* = 5.81, df = 1, 248, *P* = 0.017), with lowest infestation on IPM farms in low NDVI and highest infestation on IPPM farms in low NDVI.

**Table 2.** Interaction of IPM (no, yes), PS (no, yes), and NDVI (low, and medium) on Tephritid fruit fly infestation index using recovered puparia/kg of infested pumpkin fruits across 2 growing seasons in Machakos County, Kenya

				Puparia/kg (infestation index)	
IPM	PS	NDVI	*N*	Season 1	Season 2
No	No	Low	32	155.60 ± 88.87a	292.09 ± 142.59ab
No	Yes	Low	32	110.91 ± 35.32a	131.38 ± 57.86ab
Yes	No	Low	32	137.59 ± 78.20a	29.13 ± 19.39a
Yes	Yes	Low	32	331.33 ± 207.76a	369.49 ± 248.94b
No	No	Medium	32	17.63 ± 12.35a	92.80 ± 45.61ab
No	Yes	Medium	32	320.79 ± 153.66a	71.73 ± 38.78ab
Yes	No	Medium	32	180.71 ± 84.50a	174.42 ± 76.60b
Yes	Yes	Medium	32	249.47 ± 168.16a	57.05 ± 34.43ab

Different small letters in the same column indicate a significant difference at α = 0.05 according to the Tukey HSD test.

### Number of Fruits and Yield

**Fig. 2.**
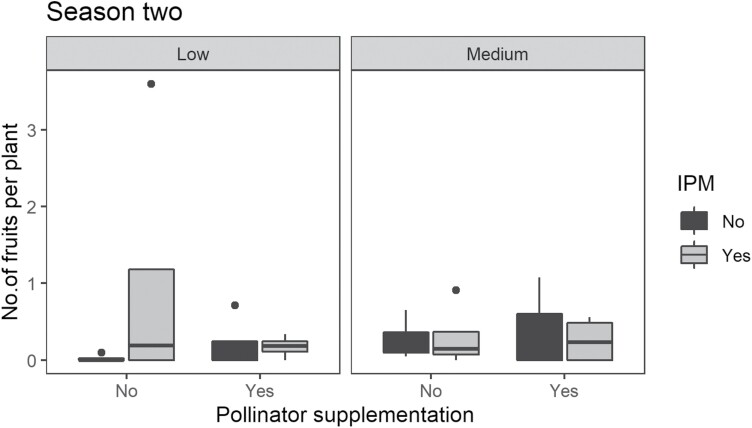
Interaction between IPM (no, yes), PS (no, yes), and NDVI (low, medium), on the number of fruits/plant harvested during the second season in Machakos County, Kenya.

During the first season, there was no significant effect of IPM (*F* = 1.46, df = 1, 7.73, *P* = 0.24), PS (*F* = 0.40, df = 1, 7.73, *P* = 0.53), NDVI (*F* = 2.96, df = 1, 7.73, *P* = 0.10), or their interaction (*F* = 0.11, df = 1, 7.73, *P* = 0.75) on the number of fruits/plant, which averaged 1.02 ± 0.32 fruits/plant (Fig. 2). In the second season, IPM significantly (*F* = 10.12, df = 1, 6.56, *P* = 0.004) influenced the number of fruits/plant, with more fruits on IPM farms (1.58 ± 1.13 fruits/plant) than on farms without IPM (0.22 ± 0.08 fruits/plant). However, the number of fruits per plant did not differ significantly across NDVI (*F* = 0.29, df = 1, 6.56, *P* = 0.60) or PS (*F* = 0.13, df = 1, 6.56, *P* = 0.72). There was no significant interaction among IPM, PS, and NDVI on the number of fruits/plant (*F* = 2.22, df = 1, 6.56, *P* = 0.15).

In the first season, there was no main effect of IPM, PS, NDVI, and their interaction (*F* ≤ 1.54, df = 1, 6.57, *P* ≥ 0.23) on yield, which averaged 475.31 ± 133.17 kg/ha across treatments. During the second season, IPM, PS, NDVI, and their interaction had no significant effect on the yield (*F* ≤ 0.29, df = 1, 9.10, *P* ≥ 0.60), which averaged 180.78 ± 59.85 kg/ha.

## Discussion

The sustainability of pumpkin production relies in part on IPM and PS to suppress key Tephritid fruit fly pests and augment pollination, ultimately increasing yield. During the study, *Z*. *cucurbitae* was reported as the key fruit fly species from the cuelure traps with minimal catches recorded for *D. bivittatus* and *D. punctatifrons*. These results are in line with findings from earlier studies, which reported high attraction of fruit flies in the genera *Bactrocera* and *Zeugodacus* to cuelure traps, with minimal attraction to fruit flies in the genus *Dacus* (Gnanvossou et al. 2017, Royer et al. 2020). In the first season, there was no effect of the treatments on the daily catches of *Z. cucurbitae*, while in the second season, *Z. cucurbitae* catches were higher in farms with no PS than in farms with PS, implying that the presence of stingless bee colonies did not increase the abundance of *Z. cucurbitae*. Contrary to our study, Toukem et al. (2022) reported an increase in *B. dorsalis* catches on avocado farms with honeybee colonies, which was attributed to the smell of honey sugar and fermentation of beebread, which attracted fruit flies (Lee et al. 2015). Furthermore, in the second season, *Z. cucurbitae* catches were highest in control farms across low NDVI, presumably due to fewer natural enemies (Copeland et al. 2006) and as reported by Mwatawala et al. (2006) for Tanzanian lowland regions. Moreover, Toukem et al. (2020) reported a higher abundance of Tephritid fruit flies on avocado in low NDVI. We found the lowest daily catches of *Z. cucurbitae* in IPPM farms in medium NDVI, but only during the second season, illustrating a synergistic effect. Unlike in our study, Toukem et al. (2022) reported no synergistic effect of IPM and PS in reduction of Tephritid fruit fly densities in avocado production systems, however, independently, IPM reduced fruit fly pest abundance. Findings from our study reported a main effect of NDVI on the catches of *D. bivittatus*, with the highest catches observed across low NDVI. This confirms the importance of presence of vegetation to reduce cucurbit-infesting fruit fly populations. Although the daily catches of *D. bivittatus* were generally lower than those of *Z. cucurbitae*, their population densities were higher in IPPM farms and lower across PS farms. Also, *D. punctatifrons* populations were higher in farms without colonies during the second season. Presumably, *D. bivittatus* and *D. punctatifrons* adults could have been attracted to the smell of honey sugar and fermentation of beebread from the *Hypotrigona* sp. colonies (Mohammad et al. 2021).

Our study reports emergence of *Z. cucurbitae*, *D. bivittatus*, *D. vertebratus*, and *D. ciliatus* from incubated pumpkin fruits in very low numbers. The low emergence could be attributed to the natural control by *Psyttalia* sp., a fruit fly parasitoid, which was documented emerging from the incubated infested pumpkin fruits. Although present in trap catches, *D. punctatifrons* was not among the fruit fly species that emerged from incubated fruits. However, other studies have reported the emergence of *D. punctatifrons* from cucurbits, in particular watermelon (Layodé et al. 2019). Surprisingly, in the first season, highest infestation was reported in IPPM farms and lowest infestation in control farms, possibly due to high attraction of adult Tephritid fruit flies to the smell of honey and fermenting beebread from the *Hypotrigona* sp. colonies in IPPM farms, which may have resulted in successful mating and oviposition. In the second season, lowest infestation level was reported in IPM farms in low NDVI, indicating that the IPM package reduced the number of fruit flies.

During the first season, the number of fruits per plant was not different across treatments, whereas in the second season, the number of fruits per plant was higher in farm plots with IPM than those without, illustrating the effectiveness of the IPM package to combat Tephritid fruit flies in cucurbits, as illustrated by Githiomi et al. (2019) and Midingoyi et al. (2019) for mango, Pecenka et al. (2021) for watermelon, and Toukem et al. (2022) for avocado. However, a higher number of fruits/plant reported in IPM farms in the second season did not translate to higher yields across the treatments. We, therefore, recommend for the IPM package to be fine-tuned for improved pumpkin yields. Introduction of *Hypotrigona* sp. hives did not significantly increase the number of pumpkin fruits/plant in both seasons, indicating that *Hypotrigona* sp. may not be an effective pumpkin flower pollinator. In contrast to this finding, a previous study reported effective pollination of *C. sativus* using *Hypotrigona gribodoi* yielding 90% seed germination (Kiatoko et al. (2021b). *Hypotrigona* sp. has a small body size of 2- to 3-mm body length (Heard, 1999), and the number of pollen attached to the body of stingless bees is positively correlated to body weight (Pangestika et al. 2017). Since a total of 1,500–2,000 pollen grains/flower are required for complete pumpkin pollination (Vidal et al. 2010), sufficient pollination may not have taken place. Additionally, the short period of anthesis (6–12 h) in pumpkin may have limited duration for pollen retrieval by stingless bees (Vidal et al. 2010). Although spinosad-based protein baits used in the study are widely incorporated in IPM programs as a replacement for organophosphates for the control of fruit flies, they have been reported to have toxic effects on Africanized honeybees and stingless bees such as *Plebeia lucii* Moore (Apidae: Meliponini) (Lopes et al. 2018, Marques et al. 2020). However, a recent study by Toukem et al. (2022) in avocado production systems in Kenya reported contradictory results, with abundant honeybee populations documented in IPM farms incorporating spinosad-based protein baits. Additionally, Koech et al. (2023) reported low spinosad contamination on pollen collected by honeybees in avocado farms across Muranga landscapes. The contamination was below the recommended EU limits and therefore not detrimental to honeybee health.

Yield in the first season was 2.6 times higher than yield in the second season, reflecting the importance of sufficient rain during the growing season. In the second season, fruit fly densities were much higher (12.39 ± 0.78 catches/trap/day) than in the first season (7.51 ± 0.45 catches/trap/day). Frequent heavy rains encountered during the first season may have attributed to the reduction in fruit fly densities, as demonstrated by Ye and Liu (2005). A previous study has indicated that fruit fly populations in avocado are affected by landscape and time factors, with population densities generally higher in lower NDVI compared to higher NDVI (Toukem et al. 2020). We reported similar findings, but only for *Z. cucurbitae* and *D. bivittatus* populations, mainly in the second season. Presumably, when high pest populations are present, lack of vegetation translates in a reduced presence of natural enemies (Bianchi et al. 2006).

This study revealed no synergistic effect between IPM and PS in suppressing Tephritid fruit fly population densities and damage. This was especially evident during the main growing season with low Tephritid fruit fly pest densities. However, in season 2, with high Tephritid fruit fly pest densities, *Z. cucurbitae* population densities were reduced in IPPM, but this reduction did not translate in reduced fruit damage or increased yield. *Hypotrigona* sp. may not be an efficient pollinator of pumpkin and we recommend testing other African stingless bees such as *M. ferruginea* and *M. bocandei* in pumpkin production systems for better pollination services and improved yields.
